# Analysis of Chaotic Behavior in a Novel Extended Love Model Considering Positive and Negative External Environment

**DOI:** 10.3390/e20050365

**Published:** 2018-05-14

**Authors:** Linyun Huang, Youngchul Bae

**Affiliations:** 1 Department of Biomedical and Electronic Engineering, Chonnam National University, Yeosu 59626, Korea; 2 Division of Electrical Electronic Communication and Computer Engineering, Chonnam National University, Yeosu 59626, Korea

**Keywords:** love model, external environment, periodic motion, Rössler type attractor, chaotic attractor

## Abstract

The aim of this study was to describe a novel extended dynamical love model with the external environments of the love story of Romeo and Juliet. We used the sinusoidal function as external environments as it could represent the positive and negative characteristics of humans. We considered positive and negative advice from a third person. First, we applied the same amount of positive and negative advice. Second, the amount of positive advice was greater than that of negative advice. Third, the amount of positive advice was smaller than that of negative advice in an external environment. To verify the chaotic phenomena in the proposed extended dynamic love affair with external environments, we used time series, phase portraits, power spectrum, Poincare map, bifurcation diagram, and the maximal Lyapunov exponent. With a variation of parameter “a”, we recognized that the novel extended dynamic love affairs with different three situations of external environments had chaotic behaviors. We showed 1, 2, 4 periodic motion, Rössler type attractor, and chaotic attractor when parameter “a” varied under the following conditions: the amount of positive advice = the amount of negative advice, the amount of positive advice > the amount of negative advice, and the amount of positive advice < the amount of negative advice.

## 1. Introduction

We live in the nonlinear and complex world, and human society is one of the world’s that can described as a complex and nonlinear system. The notion of describing human society as a complex system is derived from nonlinear dynamics, information theory, and statistical physics. Nonlinear dynamics includes neural networks, complex systems, chaotic dynamic systems, fuzzy systems, and so on. Chaotic dynamics systems have characteristics of depending on initial conditions and self-similarity. Compared to linear systems, nonlinear systems are more difficult to analyze, synthesize, and implement characteristics and behaviors.

Over the last few decades, the analysis and applications of chaotic dynamics in nonlinear systems have been widely studied in relation to various natural science disciplines including mathematics, chemistry, physics [1,2], and engineering [3,4]. However, many scholars and scientists have recently shown interest in applying these natural science models to social sciences [5] including areas such as psychology [6], family [7], addiction [8,9,10,11,12], happiness [13,14,15,16], and adult love and romantic relationships [17,18,19,20,21,22,23,24,25]. Particularly, the relationship between love affairs and romance of humans deals with different or similar contents in mathematics, biology, psychology, anthropology, sociology, cognitive and medical science, and sexology. Among these social science areas, several researchers have shown interest in finding a love model that can describe differential equations. Several models of love affairs have been proposed based on historical love affairs such as the Romeo and Juliet model [17,18,19,20,21,22,23,24,25], Laura and Petrarch model [26], and Adam and Eve model [27]. Among these love models, the model of love affairs based on Romeo and Juliet is the most commonly used one in the research of nonlinear or chaotic dynamics.

Strogatz was the first scholar to suggest the use of differential equations to study love affairs with a simple model for ill-fated romance based on Shakespeare’s “Romeo and Juliet”, using a second order linear system. In addition, Strogatz and his students studied different love affairs considered as star-crossed romances and suggested that ‘eager beaver’ and ‘cautious lover’ reference types of lovers were governed by the general linear system according to the status of parameter values [18]. Through the reference [18], he inspired many mathematicians, physicians, and engineers to pay attention to the human mind and thinking such as love, hate, happiness, unhappiness, and so on. Inspired by the study of Strogatz [18], Sprott [17] first proposed love affairs of Romeo and Juliet using a linear differential equation. He considered six statuses of romantic styles for Romeo and Juliet. He extracted a total of 36 different pairings of romantic styles according to parameter values. Since the romantic style of Romeo and Juliet was symmetric, he only suggested 21 unique dynamic statuses. At the end, he only considered four statuses among these 21 statuses. Sprott [17] also analyzed the triangle relationship of the love model and described the chaotic behaviors. 

Wauer et al. [19] proposed models of love with time-varying fluctuations and analyzed the dynamical characteristics of this model. They studied time-dependent fluctuations in both the source terms and in system parameters. However, their paper did not deal with the chaotic behaviors including time series, phase portrait, power spectrum, Poincare map, bifurcation, and Lyapunov exponent necessary to determine chaotic behaviors.

Son et al. [20] proposed a time delay effect in a love dynamical model with Hopf bifurcation and a periodic-doubling bifurcations diagram. Their paper focused on the effect of time delay on the dynamics of a love model based on a non-synergic couple. The love model proposed by Son et al. became a third order system because the time delay increased the system order. Their paper showed the time delay effect in a love dynamical model by using time series, phase portrait, and bifurcation diagram. 

Rinaldi et al. [21,22,23] proposed more realistic mathematical models for love affairs than the one proposed by Strogatz and Sprott [17,18]. Their papers were based on love stories from novels, theatre, and movies, like, Pride and Prejudice [28,29], Kathe, Jules and Jim [30], Scarlett and Rhett [31], Beauty and The Beast [32], and Roxanne and Cyrano [33] and deal with relationships of human love.

Bae et al. [24,25,34,35,36,37,38,39,40,41] proposed that the love model of Romeo and Juliet had periodic motion and chaotic behavior through time series and phase portraits. It deals with the same and different time delays [24,25]. It also applies several types of external environment such as sine wave, sine wave with Fourier series, discontinuous function, Gaussian, triangular and fuzzy membership function [34,35,36,37,38,39,40,41]. However, Bae et al. did not consider detailed relationships as external environments such as qualitative or quantitative analysis of characteristics. Rinaldi et al. [42] considered environmental stress as impulse, step and sinusoidal functions of time. It only deals with stress from outside. Most previously published papers did not demonstrate an exact chaotic phenomenon in the Romeo and Juliet model with time series, phase portrait, power spectrum, Poincare map, Lyapunov exponent, and bifurcation diagram. They demonstrated chaotic phenomena or behaviors by only using time series, phase portrait, and bifurcation diagram [24,25,34,35,36,37,38,39,40].

In social science, in order to analyze chaotic behaviors such as addiction, happiness, and love included in the Romeo and Juliet model, we require mathematical representation using ordinary differential equations or difference equations. We then need to verify these chaotic behaviors through time series, phase portrait, power spectrum, Poincare map, Lyapunov exponent, and bifurcation diagram. Since each method cannot guarantee sufficient condition, we need to apply all methods to satisfy the necessary-sufficient condition that proves chaotic behavior in the Romeo and Juliet love model. 

Therefore, this paper proposed a novel dynamic love model considering positive and negative external environments in the Romeo and Juliet story. We used sinusoidal function with different magnitude as an external environment as it could represent positive and negative characteristics of the external environment in the love model. Generally, the external environment can contain economic, psychologic, and emotional status. It mainly comes from the advice of a third party such as their parent, relatives, friends, and social network system including Twitter, Facebook, blogs, and so on. The advice can involve qualitative and quantitative characteristics and can be divided into two categories: positive and negative advice. Thus, we can represent positive and negative advice by using a sine wave function with different magnitude. To verify chaotic behavior in the proposed dynamic love model, we used time series, phase portraits, power spectrum, Poincare map, bifurcation diagram, and the maximal Lyapunov exponent. From this process, we verified the existence of chaotic behaviors including periodic doubling and periodic window for the novel extended dynamic love model with sine wave function as same and different magnitude.

## 2. Love Model of Romeo and Juliet

### 2.1. Basic Love Model of Romeo and Juliet

The basic love model proposed by Strogatz [18] and Sprott [17] can be represented with Equation (1):

(1) dR/dt=aR+bJ,dJ/dt=cR+dJ

where “a” and “b” represents Romeo’s romantic style and “c” and “d” specifies Juliet’s style. In addition, the parameter “a” describes the extent of whether Romeo is encouraged by his own feelings, and “b” is the extent of whether Romeo is encouraged by Juliet’s feelings [17]. For Juliet, the parameter “c” describes the extent of whether Juliet is encouraged by Romeo’s feelings and parameter “d” is the extent of whether Juliet is encouraged by her own feelings.

**Figure 1:**
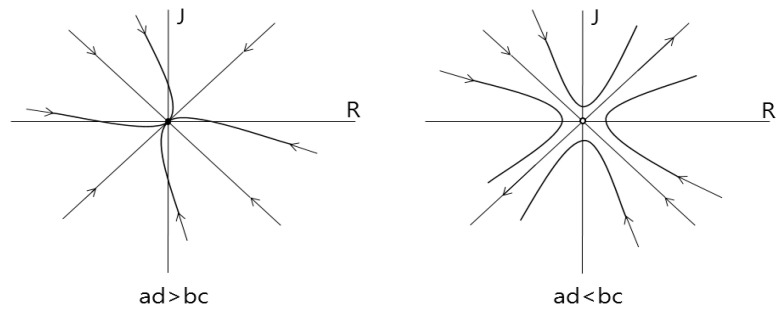
Phase portrait for the fixed point and saddle point.

Based on the equation, we know that if ad < bc, the fixed point (*R*, *J*) = (0, 0) is a saddle point and if ad > bc, it is a stable point. This means that if ad < bc, the relationship between Romeo and Juliet will either be in a love nest or in a war. If ad > bc, Romeo and Juliet will eventually become indifferent to each other as shown in Figure 1 [17].

Therefore, we have to select the first case ad < bc as the standard for the setting parameters because in this situation, Romeo and Juliet are more likely to love each other and express chaotic behavior. To satisfy the parameter condition of ad < bc of the system, we need to find a reasonable parameter range.

### 2.2. Nonlinear Love Model

Sprott [17] proposed the nonlinear love model, which can be written as Equation (2)

(2) dR/dt=aR+bJ(1−|J|),dJ/dt=cR(1−|R|)+dJ

### 2.3. Extended Love Model with an External Environment

**Figure 2:**
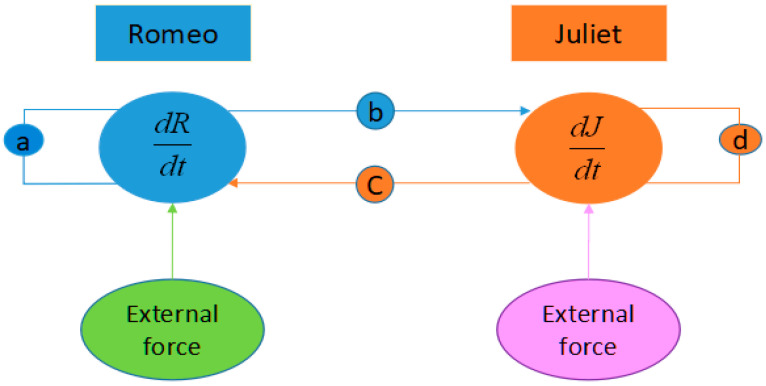
Relationship between Romeo, Juliet and the third party.

If the system is only second order, the system cannot generate the chaotic behaviors. Therefore, Equations (1) and (2) cannot obtain the chaotic phenomena. In order to generate chaotic phenomena in the dynamic love model, a third-order system containing at least one nonlinear term must be involved. As Equations (1) or (2) cannot satisfy the necessary condition generating chaotic behavior, we have to modify the formulation to generate chaotic behaviors. The extended love model proposed by Bae et al., can be written as Equation (3) including external environments, *y*(*t*) and *f*(*t*). Figure 2 shows the relationship between Romeo, Juliet, and advice from a third party. The third party can refer to parents, relatives or friends. The external environment can contain economic, psychologic, and emotional status. It also can represent the influence that comes from social network system including Twitter, Facebook, blogs, and so on.

(3) dR/dt=aR+bJ(1−|J|)+y(t), dJ/dt=cR(1−|R|)+dJ+f(t)

where *y*(*t*) and *f*(*t*) are external environment.

### 2.4. Extended Love Model with a Positive and Negative External Environment

In this paper, from Equation (3), we considered an extended love model with a positive or negative external environment from the third party of Romeo and Juliet. To represent positive and negative external environments, we used sine waves with different magnitudes to show the amount of positive and negative advice from the third party such as parents, friends, relatives, and social network system. The sine wave can be written as Equation (4)

(4) y(t)=f(t)=Asin(ωπt)+B

where A, B are the magnitude of advice, in this paper, we set A > 0. Then, the value of (A + B) and (−A + B) means the positive magnitude and negative magnitude of advice, respectively, and ω is the angular frequency which represents how often the external environment affects the love of Romeo and Juliet. To avoid complex situations, we assumed the situation as *ω* = 1. We only considered the effect of the external environment on Romeo. This means *f*(*t*) = 0.

## 3. Setting of Range of Parameter Value

To generate periodic motion and chaotic motion in Equations (3) and (4), we have to set system parameters (a, b, c, d) and external environment parameters A and B.

### 3.1. Condition of System’s Parameter Range

From the condition of Figure 1, we have to decide the range of each parameter value to satisfy ad < bc. To do this, we fixed parameters b, c, and d at −2, 1, and 1, respectively. Then we changed parameter “a” to be less than −2.

### 3.2. Condition of External Environment Parameter

**Figure 3:**
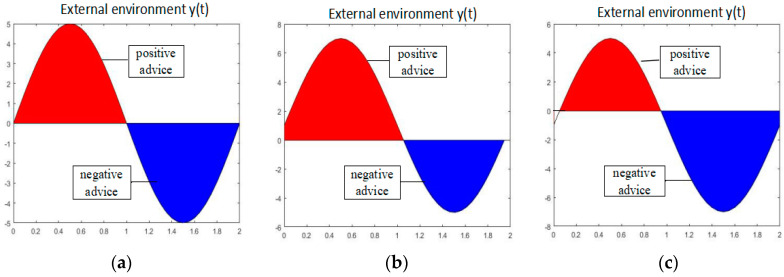
External environment situation. (**a**) Positive advice = Negative advice, (**b**) Positive advice > Negative advice, and (**c**) Positive advice < Negative advice.

From Equation (4), we can set parameters A and B that decide the magnitude of advice from the third party such as their parents, relatives, friends, and social network system. Under the condition of A > 0, (A + B) means positive advice while (−A + B) means negative advice. Figure 3 shows three external environment situations: (a) the amount of positive advice = the amount of negative advice (B = 0), (b) the amount of positive advice > that of negative advice (B > 0), and (c) the amount of positive advice < that of negative advice (B < 0).

## 4. Chaotic Behavior in the Extended Love Model with a Positive and Negative External Environment

By using Equations (3) and (4), we investigated chaotic behaviors in the love model with an external environment using the time series, phase portraits, power spectrum, Poincare map, bifurcation diagram, and the maximal Lyapunov exponent. Typically, each method does not provide sufficient conditions to determine whether the dynamics show chaotic behavior or not. However, because each method can provide a necessary-sufficient condition, it would be advantageous to use as many methods as possible to prove chaotic behaviors. Therefore, in this paper, we used all methods above-mentioned to verify the chaotic behavior of the system.

### 4.1. When b = −2, c = 1, d = 1, y(t) = 5sin(πt)

In this section, parameters A and B were set as 5 and 0, respectively. Therefore, the external environment was *y*(*t*) = 5sin(π*t*) for the love model.

#### 4.1.1. a = −5.41

**Figure 4:**
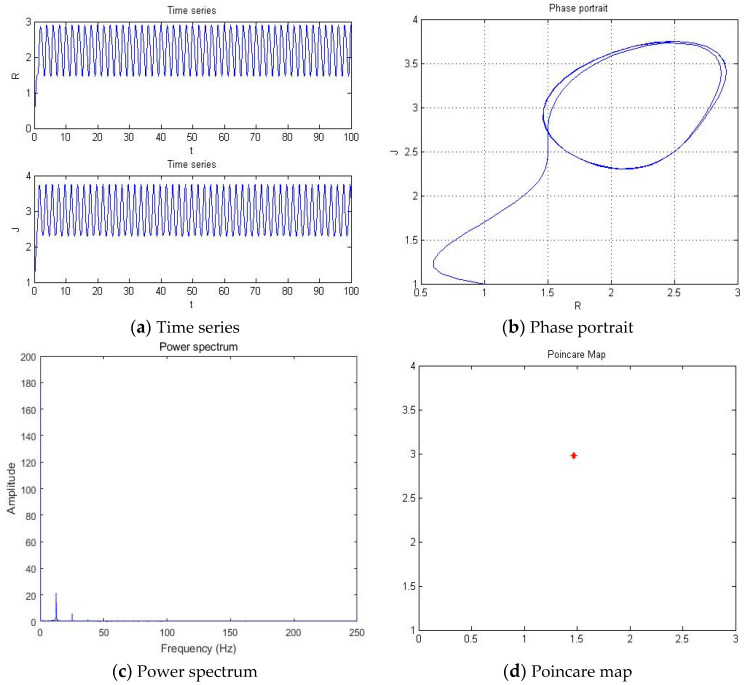
(**a**) Time series, (**b**) Phase portrait, (**c**) Power spectrum, and (**d**) Poincare map with an external environment for the Romeo and Juliet love model when parameter a = −5.41.

With parameters a = −5.41, b = −2, c = 1, d = 1, and *y*(*t*) = 5sin(π*t*) as an external environment item in Romeo, we acquired 1 periodic motion as shown in Figure 4 through the time series, phase portrait, power spectrum, and Poincare map.

The Poincare map showed 1, 2, 4, and 8 points when time series and phase portrait had 1, 2, 4, and 8 periodic motions, respectively. Meanwhile, the power spectrum showed 1, 2, 4, and 8 peaks when the time series and phase portrait had 1, 2, 4, and 8 periodic motions, respectively. From Figure 4, we can recognize that the system has one periodic motion when parameter a = −5.41.

#### 4.1.2. a = −3.451

**Figure 5:**
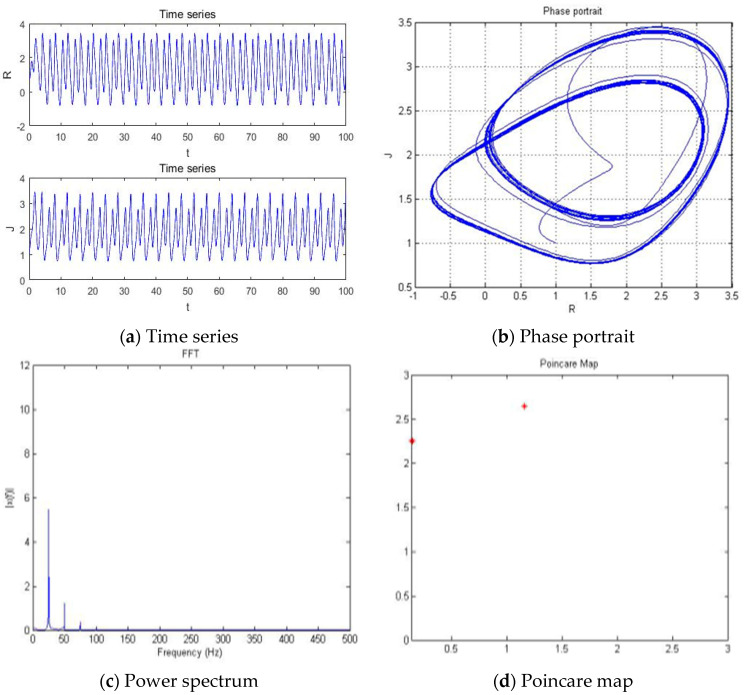
(**a**) Time series, (**b**) Phase portrait, (**c**) Power spectrum, and (**d**) Poincare map with an external environment for the Romeo and Juliet love model when parameter a = −3.451.

With parameters a = −3.451, b = −2, c = 1, d = 1, and *y*(*t*) = 5sin(π*t*) as an external environment for the extended Romeo and Juliet love model, we obtained periodic motion as shown in Figure 5 through the time series, phase portrait, power spectrum, and Poincare map.

#### 4.1.3. a = −3.406

**Figure 6:**
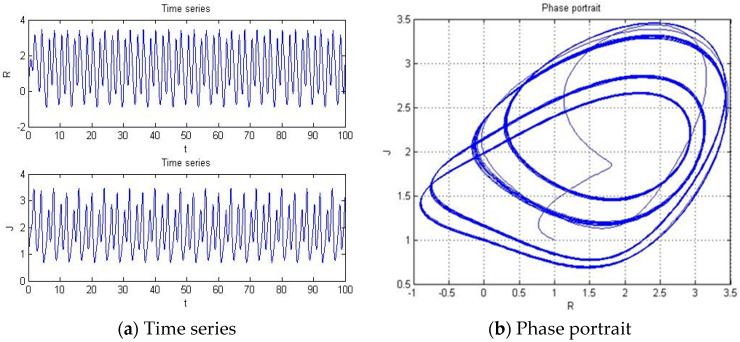

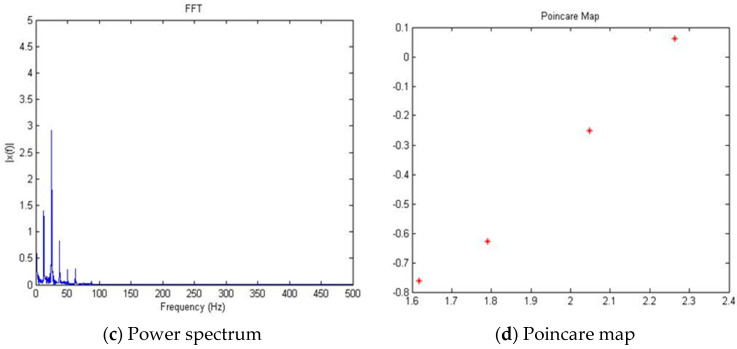
(**a**) Time series, (**b**) Phase portrait, (**c**) Power spectrum, and (**d**) Poincare map with an external environment for the Romeo and Juliet love model when parameter a = −3.406.

With parameters a = −3.406, b = −2, c = 1, d = 1, and *y*(*t*) = 5sin(π*t*) as an external environment for the extended Romeo and Juliet love model, we acquired 4 periodic motions as shown in Figure 6 through the time series, phase portrait, power spectrum, and Poincare map.

#### 4.1.4. a = −3.201

**Figure 7:**
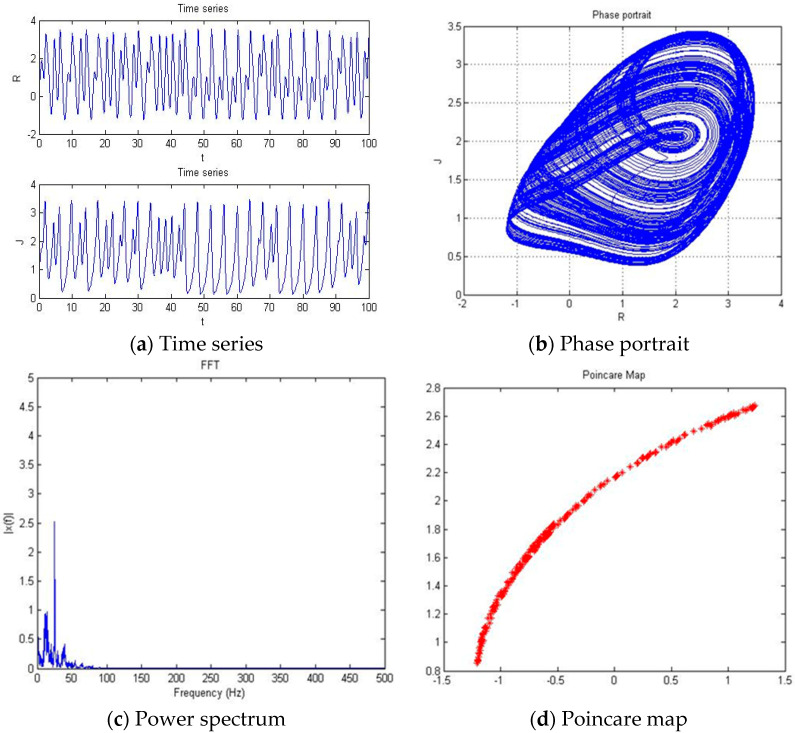
(**a**) Time series, (**b**) Phase portrait, (**c**) Power spectrum, and (**d**) Poincare map with an external environment for the Romeo and Juliet love model when parameter a = −3.201.

With parameters a = −3.201, b = −2, c = 1, d = 1, and *y*(*t*) = 5sin(π*t*) as an external environment for the extended Romeo and Juliet love model, we acquired the Rössler type attractor as shown in Figure 7 through the time series, phase portrait, power spectrum, and Poincare map.

#### 4.1.5. a = −2.561

**Figure 8:**
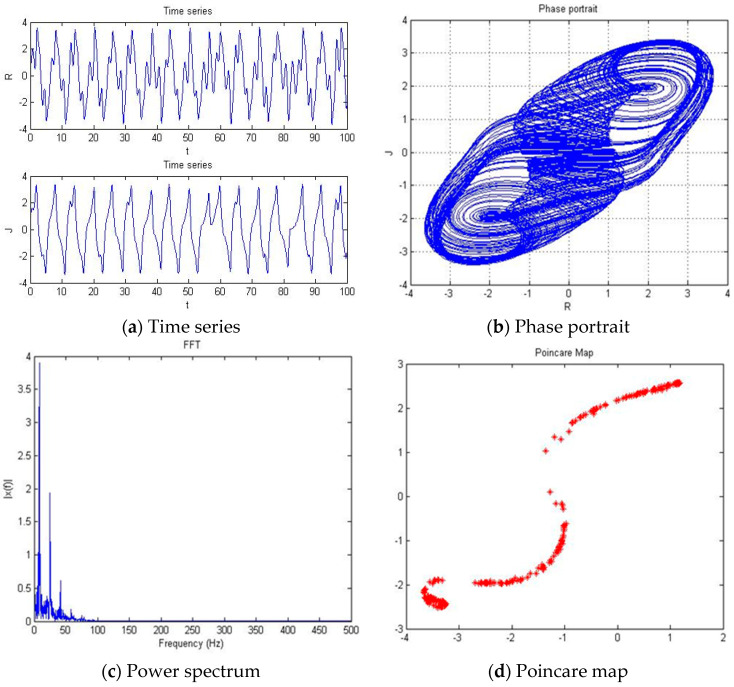
(**a**) Time series, (**b**) Phase portrait, (**c**) Power spectrum, and (**d**) Poincare map with an external environment for the Romeo and Juliet when parameter a = −2.561.

**Figure 9:**
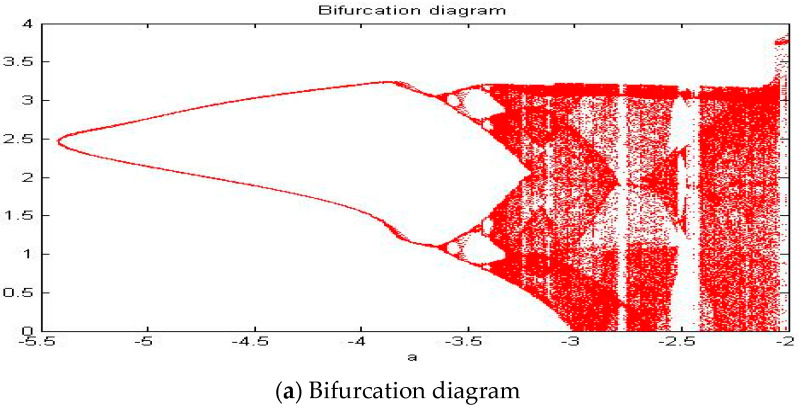

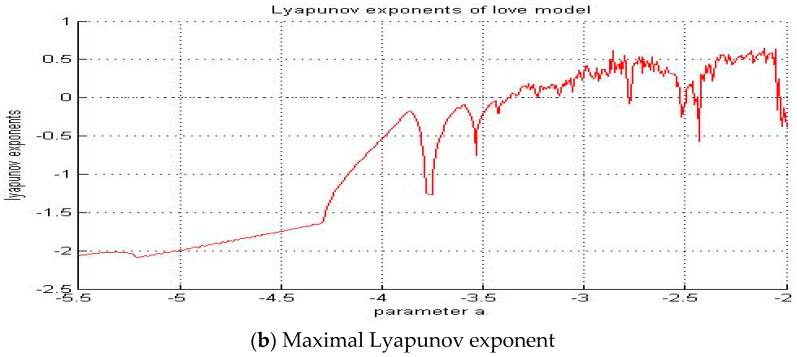
(**a**) Bifurcation diagram and (**b**) Maximal Lyapunov exponent for the Romeo and Juliet love model with an external environment.

With parameters a = −2.561, b = −2, c = 1, d = 1, and *y*(*t*) = 5sin(π*t*) as an external environment for the extended Romeo and Juliet love model, we acquired a double scroll attractor similar to Chua’s circuit as shown in Figure 8 through the time series, phase portrait, power spectrum, and Poincare map. We used the bifurcation diagram and maximal Lyapunov exponent to verify the results shown in Figure 9. Due to the limited length of this article, we only provided the time series, phase portrait, Poincare map, and power spectrum for several points.

### 4.2. When b = −2, c = 1, d = 1, y(t) = 6sin(πt) + 1

In this section, parameter A was 6 and parameter B was 1. This means that the amount of positive advice was greater than that of the negative advice. Thus, the external environment was *y*(*t*) = 6sin(π*t*) + 1 for the love model.

#### 4.2.1. a = −5.41

**Figure 10:**
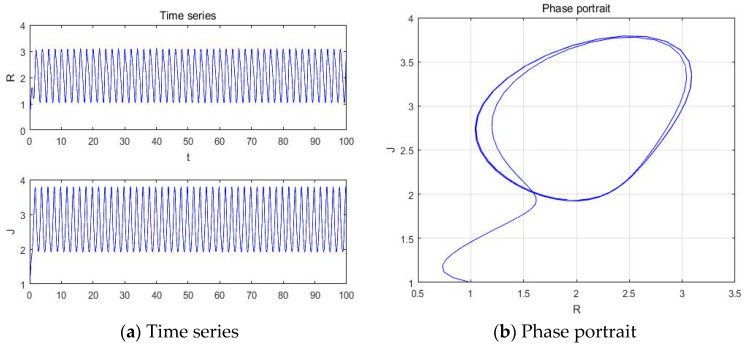

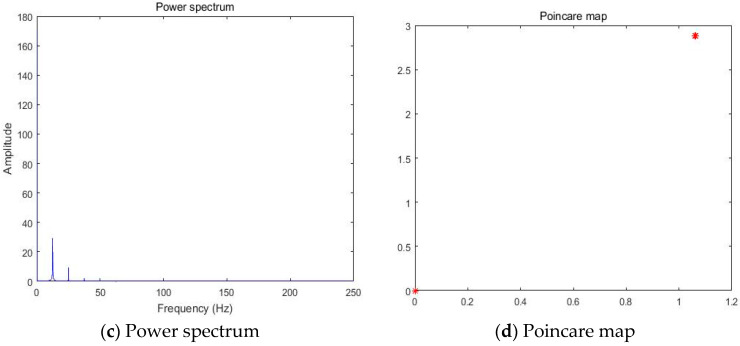
(**a**) Time series, (**b**) Phase portrait, (**c**) Power spectrum, and (**d**) Poincare map for the Romeo and Juliet love model with an external environment when parameter a = −5.41.

With parameters a = −5.41, b = −2, c = 1, d = 1, and *y*(*t*) = 6sin(π*t*) + 1 as an external environment for the extended Romeo and Juliet love model, we acquired 1 periodic motion as shown in Figure 10 through the time series, phase portrait, power spectrum, and Poincare map.

#### 4.2.2. a = −3.451

**Figure 11:**
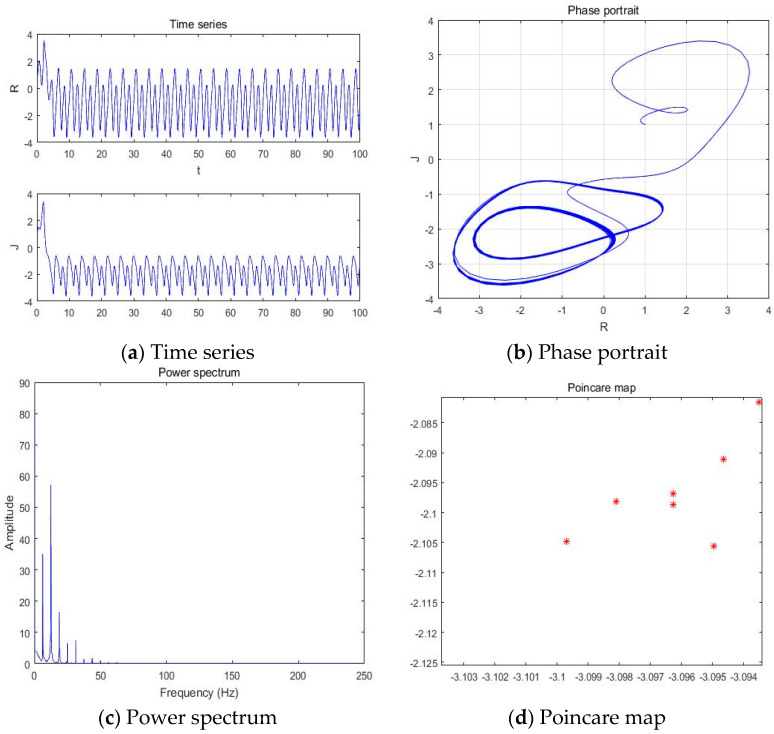
(**a**) Time series, (**b**) Phase portrait, (**c**) Power spectrum, and (**d**) Poincare map for the Romeo and Juliet love model with an external environment when parameter a = −3.451.

With parameters a = −3.451, b = −2, c = 1, d = 1, and *y*(*t*) = 6sin(π*t*) + 1 as an external environment for the extended Romeo and Juliet love model, the results of the time series, phase portrait, power spectrum, and Poincare map are shown in Figure 11.

#### 4.2.3. a = −3.406

**Figure 12:**
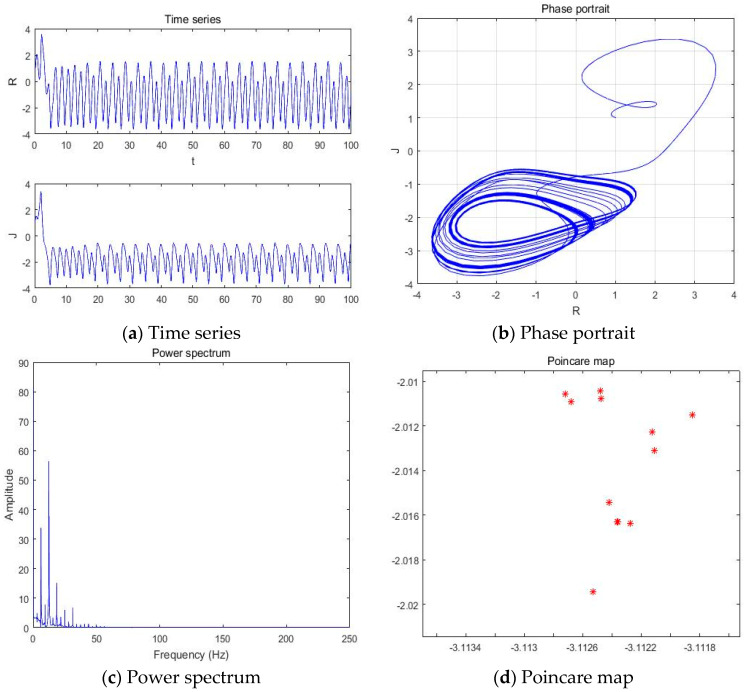
(**a**) Time series, (**b**) Phase portrait, (**c**) Power spectrum, and (**d**) Poincare map for the Romeo and Juliet love model with an external environment when parameter a = −3.406.

When we have a = −3.406, b = −2, c = 1, d = 1 as parameters and *y*(*t*) = 6sin(π*t*) + 1 as an external environment for the extended Romeo and Juliet love model, the results of the time series, phase portrait, power spectrum, and Poincare map are shown in Figure 12.

#### 4.2.4. a = −3.201

**Figure 13:**
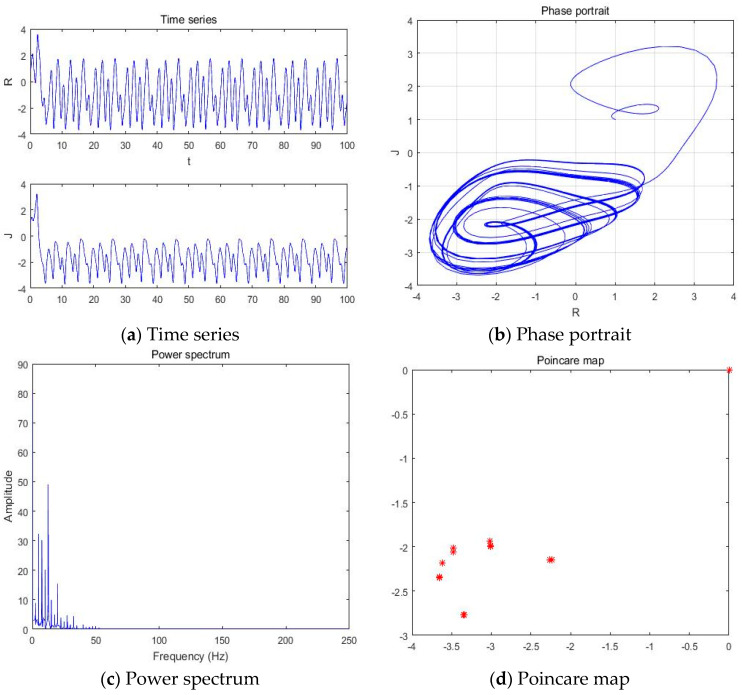
(**a**) Time series, (**b**) Phase portrait, (**c**) Power spectrum, and (**d**) Poincare map for the Romeo and Juliet love model with an external environment when parameter a = −3.201.

When we have a = −3.201, b = −2, c = 1, d = 1 as parameters and *y*(*t*) = 6sin(π*t*) + 1 as an external environment for the extended Romeo and Juliet love model, the results of the time series, phase portrait, power spectrum, and Poincare map are shown in Figure 13.

#### 4.2.5. a = −2.561

**Figure 14:**
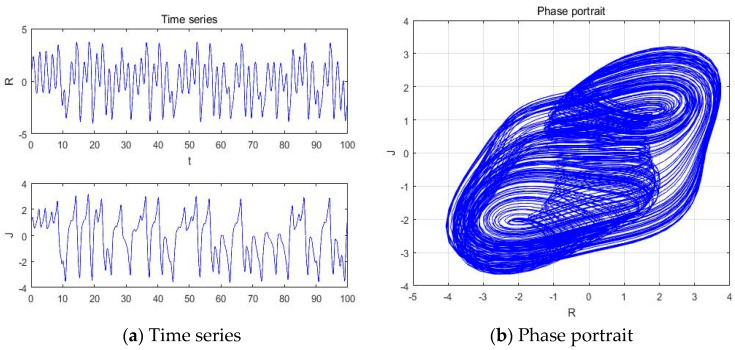

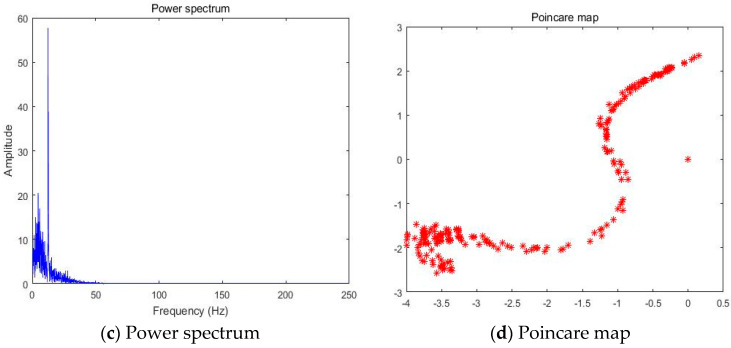
(**a**) Time series, (**b**) Phase portrait, (**c**) Power spectrum, and (**d**) Poincare map for the Romeo and Juliet love model with an external environment when parameter a = −2.561.

**Figure 15:**
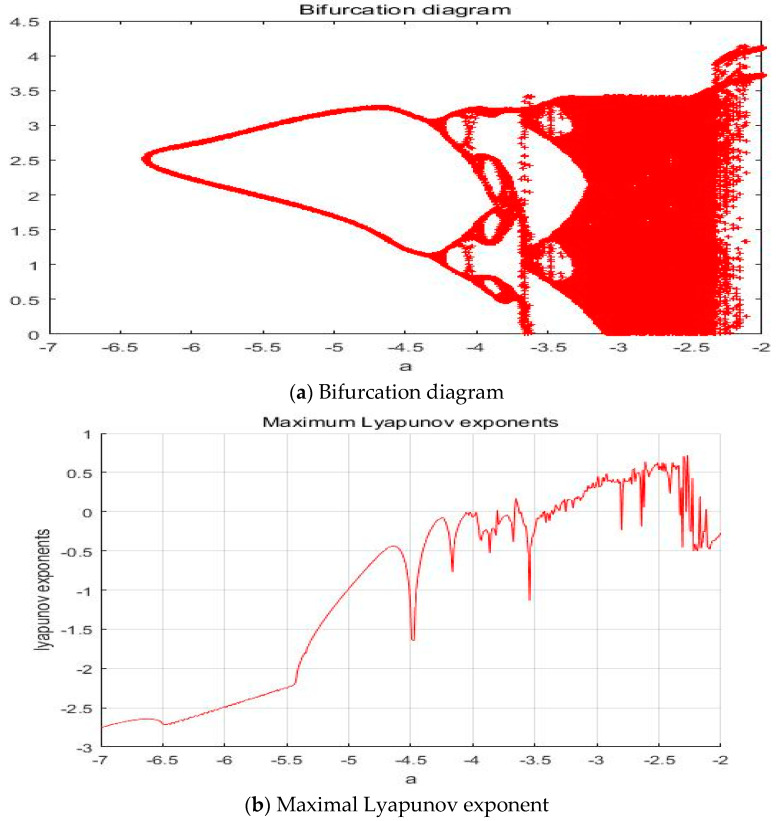
(**a**) Bifurcation diagram and (**b**) Maximal Lyapunov exponent for the Romeo and Juliet love model with an external environment.

When we had a = −2.561, b = −2, c = 1, d = 1 as parameters and *y*(*t*) = 6sin(π*t*) + 1 as an external environment for the extended Romeo and Juliet love model, we acquired chaotic motion as shown in Figure 14 through the time series, phase portrait, power spectrum, and Poincare map. We used the bifurcation diagram and maximal Lyapunov exponent to verify the results shown in Figure 15.

### 4.3. When b = −2, c = 1, d = 1, y(t) = 6sin(πt) − 1

In this section, parameter A was 6 and parameter B was −1. This means that the amount of positive advice was smaller than that of the negative advice. The external environment for the love model was *y*(*t*) = 6sin(π*t*) − 1.

#### 4.3.1. a = −5.41

**Figure 16:**
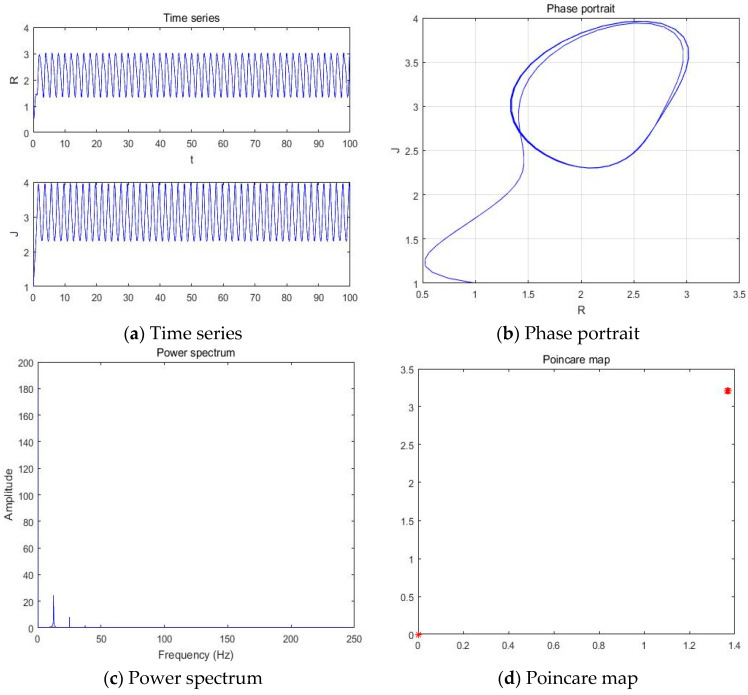
(**a**) Time series, (**b**) Phase portrait, (**c**) Power spectrum, and (**d**) Poincare map for the Romeo and Juliet love model with an external environment when parameter a = −5.41.

When we had a = −5.41, b = −2, c = 1, d = 1 as parameters and *y*(*t*) = 6sin(π*t*) − 1 as an external environment for the extended Romeo and Juliet love model, we acquired 1 periodic motion as shown in Figure 16 through the time series, phase portrait, power spectrum, and Poincare map.

#### 4.3.2. a = −3.451

**Figure 17:**
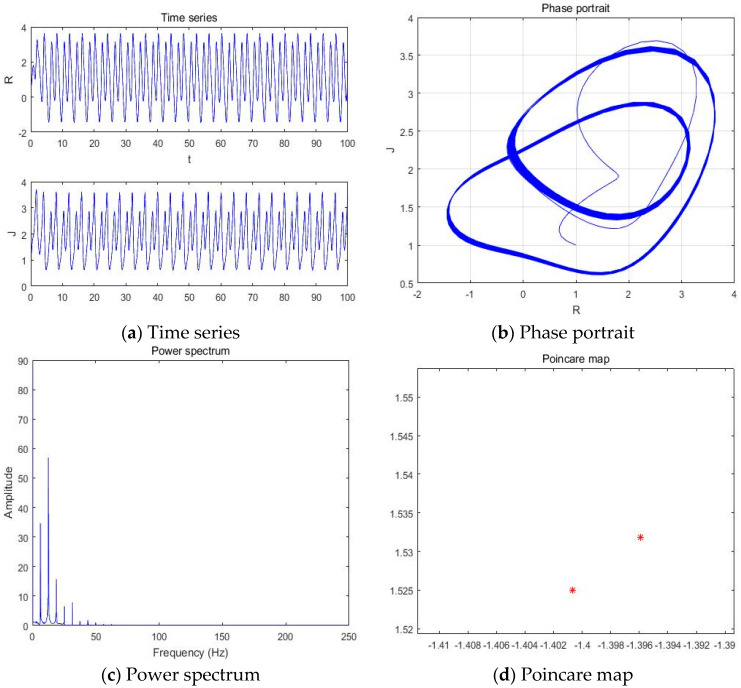
(**a**) Time series, (**b**) Phase portrait, (**c**) Power spectrum, and (**d**) Poincare map for the Romeo and Juliet love model with an external environment when parameter a = −3.451.

When we have a = −3.451, b = −2, c = 1, d = 1 as parameters and *y*(*t*) = 6sin(π*t*) − 1 as an external environment for the extended Romeo and Juliet love model, the results of the time series, phase portrait, power spectrum, and Poincare map are shown in Figure 17.

#### 4.3.3. a = −3.406

**Figure 18:**
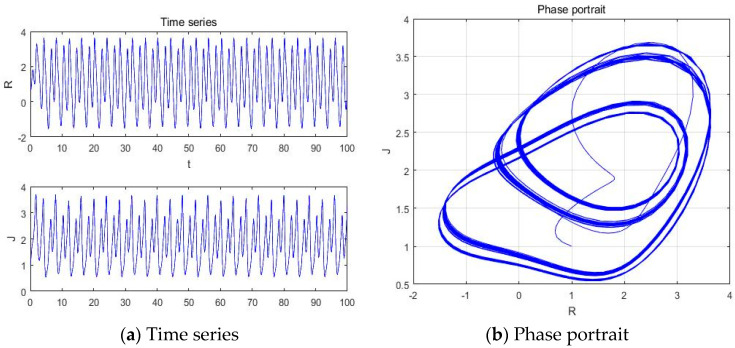

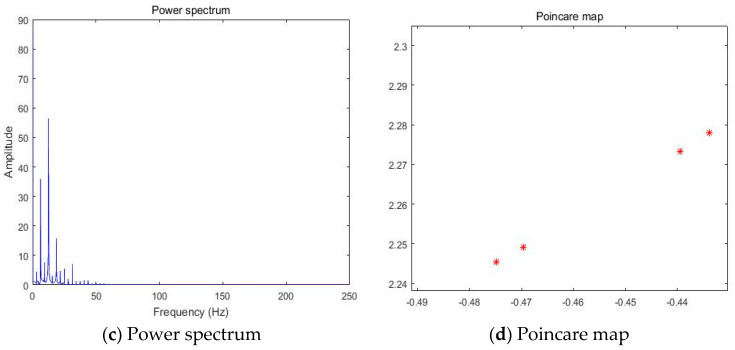
(**a**) Time series, (**b**) Phase portrait, (**c**) Power spectrum, and (**d**) Poincare map for the Romeo and Juliet love model with an external environment when parameter a = −3.406.

When we have a = −3.406, b = −2, c = 1, d = 1 as parameters and *y*(*t*) = 6sin(π*t*) − 1 as an external environment for the extended Romeo and Juliet love model, the results of the time series, phase portrait, power spectrum, and Poincare map are shown in Figure 18.

#### 4.3.4. a = −3.201

**Figure 19:**
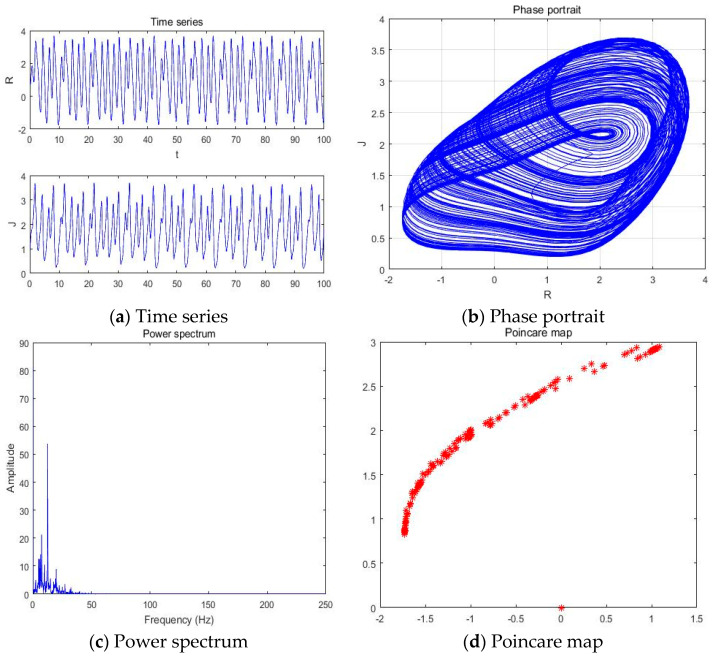
(**a**) Time series, (**b**) Phase portrait, (**c**) Power spectrum, and (**d**) Poincare map for the Romeo and Juliet love model with an external environment when parameter a = −3.201.

When we have a = −3.201, b = −2, c = 1, d = 1 as parameters and *y*(*t*) = 6sin(π*t*) − 1 as an external environment for the extended Romeo and Juliet love model, the results of the time series, phase portrait, power spectrum, and Poincare map are shown in Figure 19.

#### 4.3.5. a = −2.561

**Figure 20:**
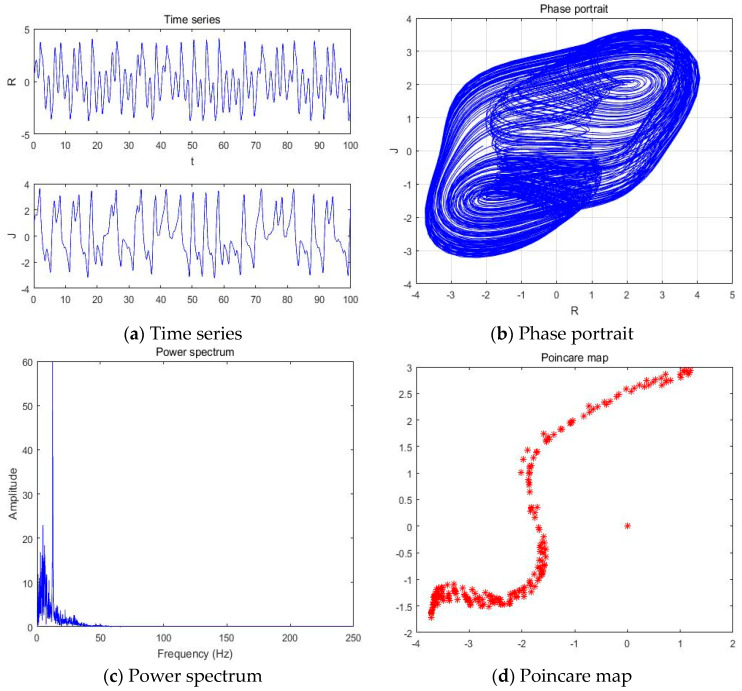
(**a**) Time series, (**b**) Phase portrait, (**c**) Power spectrum, and (**d**) Poincare map for the Romeo and Juliet love model with an external environment when parameter a = −2.561.

**Figure 21:**
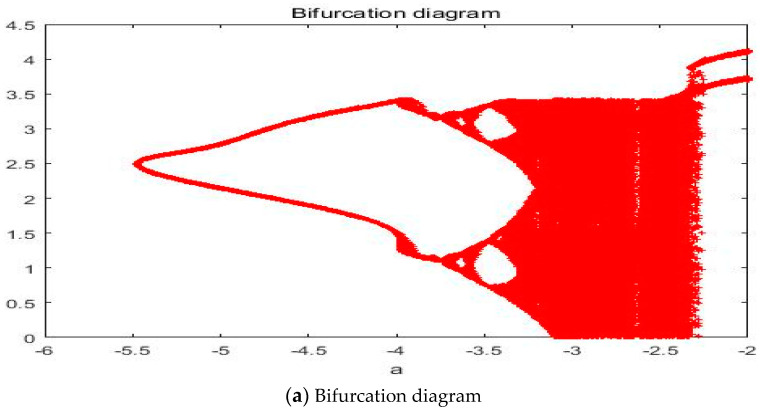

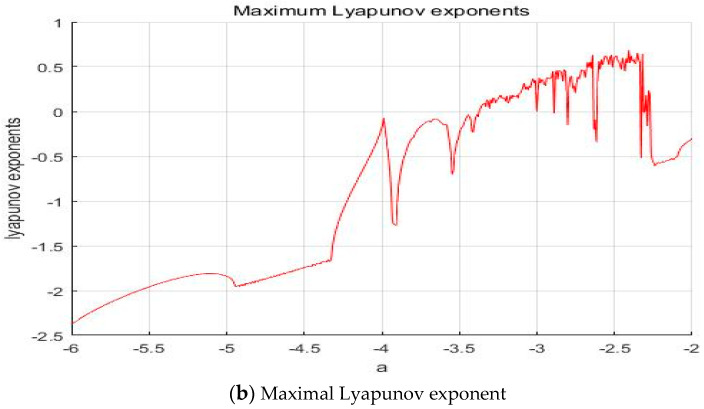
(**a**) Bifurcation diagram and (**b**) Maximal Lyapunov exponent for the Romeo and Juliet with an external environment.

When we had a = −2.561, b = −2, c = 1, d = 1 as parameters and *y*(*t*) = 6sin(π*t*) − 1 as an external environment for the extended Romeo and Juliet love model, we obtained chaotic motion as shown in Figure 20 through the time series, phase portrait, power spectrum and Poincare map. We used the bifurcation diagram and maximal Lyapunov exponent to verify the results shown in Figure 21.

## 5. Conclusions

In this paper, we proposed a novel extended dynamic love model with an external environment in the Romeo and Juliet love story. The sinusoidal function could represent positive and negative characteristics of humans, and we used sinusoidal function as an external environment to make the novel extended dynamic love model. In order to generate chaotic behavior in Romeo and Juliet, we selected parameters to satisfy condition ad < bc. We fixed parameters b, c, and d at −2, 1, and 1, respectively. Thus, parameter “a” needs to satisfy a < −2. To verify chaotic behavior in the proposed dynamic love model with external environments, we considered three cases: the amount of positive advice = that of negative advice (P = N), the amount of positive advice is greater than that of negative advice (P > N), and the amount of positive advice is smaller than that of negative advice (P < N). Then we used the time series, phase portraits, power spectrum, Poincare map, bifurcation diagram, and maximal Lyapunov exponent. When we applied these three cases as the external environment, we obtained 1, 2, 4 periodic motion at parameter a = −5.41, a = −3.451, and a = −3.406, respectively. We also acquired the Rössler type attractor and chaotic attractor when parameter a = −3.201 and a = −2.561, respectively. Results for P = N, P > N, P < N through the time series, phase portrait, power spectrum, and Poincare map were slightly different. Particularly, the bifurcation diagram showed the most different point among the three different external environments. This means that the type of negative or positive advice affects the love status between Romeo and Juliet as external environments.

As the love situations of men and women can be affected by the surrounding environment such as wording and advice from a third party, in real life, we need to select our wording and advice carefully for men and women who are loved ones.
